# Copy Number Analysis in a Large Cohort Suggestive of Inborn Errors of Immunity Indicates a Wide Spectrum of Relevant Chromosomal Losses and Gains

**DOI:** 10.1007/s10875-022-01276-8

**Published:** 2022-04-29

**Authors:** Rensheng Wan, Maximilian Schieck, Andrés Caballero-Oteyza, Winfried Hofmann, Alexis Virgil Cochino, Anna Shcherbina, Roya Sherkat, Clarisse Wache-Mainier, Anita Fernandez, Marc Sultan, Thomas Illig, Bodo Grimbacher, Michele Proietti, Doris Steinemann

**Affiliations:** 1 Department of Human Genetics, Hannover Medical School, Hannover, Germany; 2 RESIST-Cluster of Excellence 2155, Hannover Medical School, Hannover, Germany; 3 Institute for Immunodeficiency, Center for Chronic Immunodeficiency, Medical Center, Faculty of Medicine, Albert-Ludwigs-University, Freiburg, Germany; 4 Institute for Mother and Child Health, Bucharest, Romania; 5 Dmitry Rogachev National Medical and Research Center for Pediatric Hematology, Oncology, Immunology, Moscow, Russia; 6 Acquired Immunodeficiency Research Center, Isfahan University of Medical Sciences, Isfahan, Iran; 7 Novartis Institutes for BioMedical Research, Novartis Pharma AG, Basel, Switzerland; 8 Hannover Unified Biobank, Hannover Medical School, Hannover, Germany; 9 Clinic for Rheumatology and Clinical Immunology, Center for Chronic Immunodeficiency (CCI), Medical Center, Faculty of Medicine, Albert-Ludwigs-University, Freiburg, Germany; 10 DZIF – German Center for Infection Research, Satellite Center Freiburg, Freiburg, Germany; 11 CIBSS – Centre for Integrative Biological Signalling Studies, Albert-Ludwigs-University, Freiburg, Germany; 12 Department of Rheumatology and Clinical Immunology, Hannover Medical School, Hannover, Germany

**Keywords:** Primary immunodeficiency, Inborn errors of immunity, WES, CNV, SNV

## Abstract

Inborn errors of immunity (IEI) are genetically driven disorders. With the advancement of sequencing technologies, a rapidly increasing number of gene defects has been identified, thereby mirroring the high heterogeneity in immunological and clinical presentations observed in patients. However, for a large majority of patients, no causative single nucleotide variant (SNV) or small indel can be identified using next-generation sequencing. First studies have shown that also copy number variants (CNVs) can cause IEI. Unfortunately, CNVs are not well examined in many routine diagnostic settings and the aim of this study was to assess the number of clinically relevant chromosomal losses and gains in a large cohort. We identified a total of 20 CNVs using whole exome sequencing data of a cohort of 191 patients with a suspected IEI. A definite molecular diagnosis could be made in five patients (2.6%), including pathogenic deletions affecting *ICOS*, *TNFAIP3*, and 22q11.2. CNVs of uncertain significance were observed in fifteen patients (7.9%), including deletions of 11q22.1q22.3 and 16p11.2 but also duplications affecting entire or parts of genes previously associated with IEI. Importantly, five patients carrying a CNV of uncertain significance also carried pathogenic or likely pathogenic SNVs (*PIK3R1*, *NFKB1*, *NLRC4*, *DOCK2*), or SNVs of unknown significance (*NFKB2*). This cooccurrence of SNVs and CNVs suggests modifying effects in some patients, and functional follow-up is warranted now in order to better understand phenotypic heterogeneity. In summary, the diagnostic yield of IEI can be increased substantially by evaluating CNVs, which allows an improved therapeutic management in those patients.

**Supplementary Information:**

The online version contains supplementary material available at 10.1007/s10875-022-01276-8.

## Introduction

Inborn errors of immunity (IEI) are clinical conditions presenting with phenotypes of either susceptibility to viral, bacterial, or other infections or immune dysregulation such as autoinflammation or autoimmunity, or a combination thereof [1, 2]. High heterogeneity in immunological and clinical presentations exists for these genetic disorders, and the number of genetic loci associated with IEI is constantly increasing with more than 416 different monogenic defects so far, as defined by the International Union of Immunological Societies (IUIS) [3, 4]. The evolving understanding about the spectrum of affected genes and associated phenotypes caused a change in nomenclature from “primary immunodeficiency” (PID) to “inborn errors of immunity” as it became apparent that not only a mere immune deficiency but also a profound dysregulation of the immune system is caused by a single mutation in many cases. Modern gene panel sequencing or even whole exome or whole genome sequencing have been key drivers in these discoveries and are now used in many diagnostic workflows. Nevertheless, a definite genetic diagnosis is commonly made in fewer than 30% of patients with a suspected monogenic IEI. This percentage is even lower in some patient subgroups, for example, in patients with adult age at diagnosis and/or no family history of immunodeficiency [5, 6]. Importantly, this lack of proper genetic diagnoses is likely to impede an optimal therapeutic management in many patients. The low diagnostic yield might be explained by the fact that even identical variants within the same family can show extensive phenotypic heterogeneity, thereby suggesting the role of modifier genes, epigenetic regulation, and environmental factors [7]. At the same time, genetic aberrations other than single nucleotide variants (SNVs) or small indels may be missed by standard sequencing approaches. Recent studies have pointed out that the yield of genetic diagnoses is improved by taking copy number variants (CNVs) into account [8, 9]. The aim of this study was to identify CNVs with a clinical relevance in a large cohort of 191 patients with a suspected IEI.

## Materials and Methods

### Patient Cohort

A cohort of 191 patients with a suspected IEI (103 females, 88 males) was recruited at the Center for Chronic Immunodeficiency (CCI) in Freiburg, Germany, including 14 samples assigned for genetic testing from abroad. This cohort was collected with a bias towards complex cases of antibody deficiency and immune dysregulation, initially selected to identify additional patients with Activated PI3 Kinase Delta Syndrome (APDS). Clinical diagnoses were based on the European Society of Immunodeficiencies (ESID) criteria, and detailed longitudinal clinical symptom data was available for all patients [10]. We included 146 cases with common variable immunodeficiency (CVID) (76%) and 45 cases with varying immune-related conditions (Table S1). The age at diagnosis ranged from 0.42 to 79.05 years. All patients or legal guardians provided written informed consent for further investigation into the genetic basis of the disease in compliance with the guidelines of the ethical review committee of the Albert-Ludwigs-University (ethics committee vote no. 295/13 version 200,149, Freiburg, Germany).

### Whole Exome Sequencing Data

Whole blood samples of patients were collected in EDTA tubes, and isolation of genomic DNA (gDNA) was performed as described previously [11]. Sequencing libraries were prepared using the NuGEN Ovation Ultralow System V2 protocol according to the manufacturer’s guidelines (user guide M01380 v1, NuGEN Technologies, San Carlos, CA) combined with the NuGEN Ovation Target Capture Module (user guide M01291 v6) and the SureSelect^XT^ Target Enrichment System protocol (user guide G7530-90,000 vB5, Agilent Technologies, Santa Clara, CA). DNA was fragmented to an approximately 300 bp target size. Libraries were indexed and pooled in equimolar ratio for creating multiplexes. Multiplexes were further enriched for the exonic plus regions using the Agilent Technologies SureSelect^XT^ Target Enrichment System protocol (design ID C0730381; whole exome with extra custom enhanced coverage of all exons of the *PI3Kδ* and *PI3Kγ* genes). Prior, during, and after library preparation, the quality and quantity of gDNA and/or libraries were evaluated applying Qubit fluorometer 2.0 with dsDNA HS Assay Kit (Invitrogen, Carlsbad, CA) and Tapestation 4200 with D1000 kit (Agilent Technologies). Whole exome libraries were sequenced at Novartis sites on an Illumina HiSeq 2500 instrument at 2 × 76 bases + 9 bases index read length with HiSeq 2500 chemistry (product FC-401–4003, Illumina, San Diego, CA). Sequencing was performed following the manufacturer’s instructions.

### Sequence Variant Analysis

Independent from this study, whole exome sequencing (WES) data has been screened for pathogenic SNVs in genes related to IEI (unpublished data). For the here presented study, only samples carrying a potentially clinically meaningful CNV were additionally analyzed for SNVs using the medical genetics Sequence Analysis Pipeline (megSAP, v.0.2–8-ga9d80c2) for next-generation sequencing (NGS) data analysis [12]. SNVs were filtered for location in exonic and splice site regions of genes related to IEI (according to the IUIS IEI classification list of *n* = 416 genetic loci as of December 2019) [13], and a minor allele frequency (MAF) of ≤ 1% in the 1000 Genomes project database or the genome aggregation database (gnomAD). Synonymous and intronic variants were excluded from analysis. Variant annotation was performed with Alamut Visual, version 2.12 (Interactive Biosoftware, Rouen, France), and variants were visualized with the Integrative Genomics Viewer (IGV), version 2.5.3 [14]. Missense variants were analyzed in silico using Alamut Visual with the implemented tools, Align GVGD, SIFT, MutationTaster, and PolyPhen2. Splice analyses were performed with SSF, MaxEnt, NNSPLICE, and GeneSplicer. Splicing effects were considered if at least two tools provided evidence for an impact on splicing using the following thresholds: SSF ≥ 70, MaxEnt ≥ 0, NNSPLICE ≥ 0.4, and GeneSplicer ≥ 0. Datasets such as ClinVar (https://www.ncbi.nlm.nih.gov/clinvar), HGMD Professional (Qiagen, Hilden, Germany), the phyloP-value (deleterious threshold > 1.6), and the CADD score (deleterious threshold ≥ 20) were also used to classify variants. The interpretation of sequence variants followed the standards and guidelines of the American College of Medical Genetics and Genomics (ACMG) [15]. SNVs were named according to HGVS nomenclature (http://varnomen.hgvs.org).

### Copy Number Detection from WES Data

CNVs were called from WES data using the ClinCNV algorithm [16]. ClinCNV (v.1.16.2) is part of megSAP and suitable to call germline and somatic CNVs from whole exome and whole genome sequencing data. ClinCNV detects CNV changes by calculating read depth alterations, determined by the comparison with similar sequence coverage profiles within the same cohort. CNVs were filtered using the software GSvar (v.2020_03) with the following filter criteria: log-likelihood ≥ 40.00, *q*-value < 0.05, and exons affected ≥ 2 [17]. Additionally, only CNVs affecting a genetic locus related to IEI (as defined above) were considered for further evaluation. IGV was used for visualization of CNVs [14]. In order to identify potentially clinically meaningful variants, all CNVs were manually reviewed for the presence in the Database of Genomic Variants (DGV) or the gnomAD structural variant callset and excluded from further analysis if indicated to overlap with regions frequently affected by copy number variation (MAF ≥ 0.005) [18, 19].

### SNP Array Analysis

Independent validation of CNVs identified from WES data was achieved using the CytoScan XON SNP array (Life Technologies, Darmstadt, Germany), which allows for exon-level copy number detection. Processing of genomic DNA and arrays were carried out according to the user guide (publication number 703456). All data analysis was performed with the chromosome analysis suite (ChAS, Life Technologies).

### Interpretation of CNVs

CNVs were scored for pathogenicity according to the technical standards for the interpretation of constitutional CNVs established by the ACMG and the Clinical Genome Resource (ClinGen) [20, 21]. In order to determine if genes affected by CNVs belong to certain functional classes or functional pathways, an over-representation analysis was performed. Using the web-based tool g:GOSt (accessed via https://biit.cs.ut.ee/gprofiler/gost), CNVs validated in SNP array analysis were analyzed against the background list of *n* = 416 genetic loci related to IEI.

### Optical Genome Mapping

The integrity of genomic loci showing a copy number gain was assessed by optical genome mapping (OGM) [22]. In brief, for isolation of ultra-high molecular weight genomic DNA (UHMW gDNA) approximately 1 × 10^7^ PBMCs in 1 ml freezing medium (90% FBS/10% DMSO) were thawed in 37 °C water bath, transferred into 500 µl cold DNA stabilizing buffer (Bionano Genomics, San Diego, CA), centrifuged (2,200 rcf, 4 °C, 2′), supernatant removed, cell pellet washed with 1 ml cold DNA stabilizing buffer, centrifuged (2,200 rcf, 4 °C, 2′), and supernatant removed. Cell pellets were resolved in 40 µl room temperature DNA stabilizing buffer, and the cell number was assessed on a Countess II cell counter (Life Technologies). Afterwards, 2 × 10^6^ cells in 40 µl DNA stabilizing buffer were used in the manufacturer’s DNA isolation and DNA labeling protocols (doc. #30,268, #30,206). Labeled DNA was loaded on a Saphyr G2.3 chip and imaged on a Saphyr instrument (Bionano Genomics) with a target throughput of 2,000 Gbp. For de novo assembly of the genome data was filtered to 500 Gbp with a minimum molecule length of 300 kb (Bionano Tools v.1.6.1). Detection of CNVs was carried out with Bionano Access v.1.6.1. OGM has a limitation to detect small tandem duplications (TDs) (i.e., the TD carries an insufficient number of fluorescent labels). Therefore, small insertions detected by OGM were interpreted as TD if comparable in size and location to duplications determined in SNP array analysis.

## Results

### Copy Number Variant Analysis

Results of CNV detection from sequencing data and subsequent validation using SNP array are summarized in Fig. S1. In total, 251 regions (i.e., potential CNVs) affecting IEI-associated genes were called from WES data of 191 IEI patients [13]. Inferior data quality causing a high number of potentially false CNV calls was observed in four samples, which were excluded from further analysis (*n* = 91 CNVs). The *IGH@* locus presented high inter-sample variability and was also excluded from analysis in this study (*n* = 75 CNVs). Manual curation against the DGV and the gnomAD structural variant callset led to further exclusion of *n* = 51 CNVs, which overlapped with known CNVs in the general population. This reduced the number of potential CNVs detected from WES data to *n* = 34 in 29 IEI samples, which were subjected to SNP array analysis for independent validation (patient-005 and patient-060 carrying a well-described *ICOS* founder variant were excluded from validation) [23]. In the end, 20 CNVs in 20 different IEI samples were shown to be true alterations as summarized in Table 1 and Fig.S1 (whereas the other 14 were false positives). Losses of chromosomal material (deletions) were detected in eleven samples and gains of chromosomal material (duplications) in nine samples. CNVs varied in size from 1.2 kb to 8.7 Mb, with a median size of 39.1 kb (sizes of CNVs are based on SNP array analysis). Over-representation analysis indicated no clustering of CNVs to genes of certain functional classes or functional pathways.

**Table 1:**
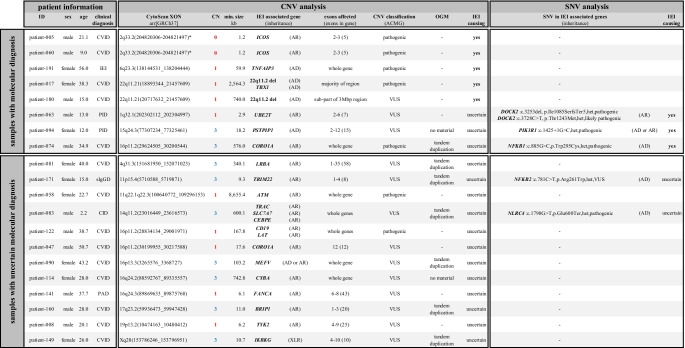
Copy number and single nucleotide variants observed in the IEI cohort. A total of 20 CNVs were identified and validated in 20 different patients. Subsequently, these 20 patients were analyzed for SNVs in IEI associated genes. The upper part of the table summarizes samples with a plausible molecular diagnosis based on a CNV or SNV with regard to the phenotype of the patient. The lower part of the table summarizes samples with an uncertain molecular diagnosis showing either a less clear correlation with regard to the observed phenotype or presenting a heterozygous event in a gene with a so far known autosomal recessive inheritance

*AD* autosomal dominant, *AR* autosomal recessive, *CID* combined immunodeficiency, *CN* copy number, *PAD* predominantly antibody deficiency, *sIgGD* selective IgG deficiency disease, *VUS* variant of uncertain significance, *XLR* X-linked recessive

### Regions of Copy Number Loss

**Fig. 1:**
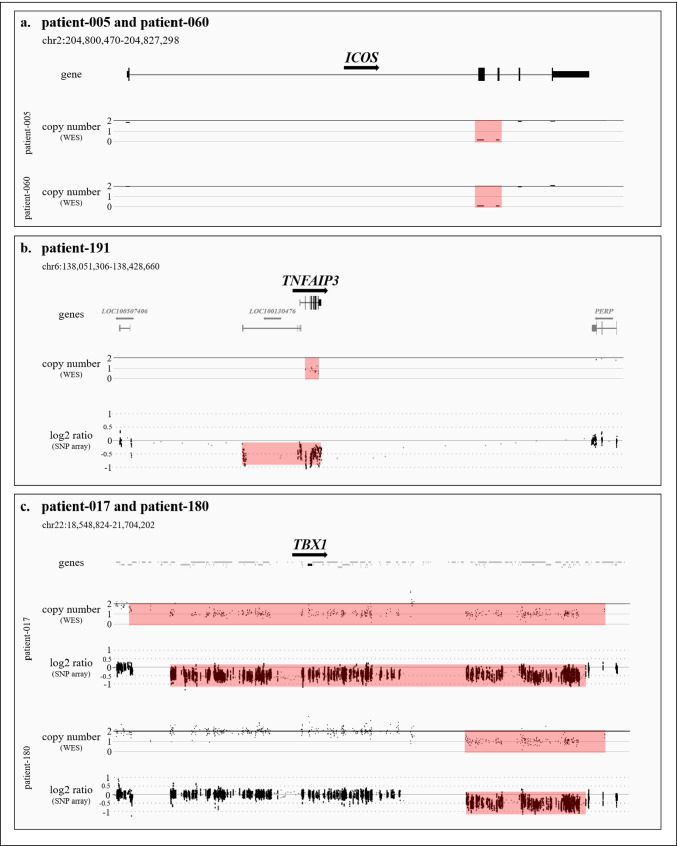
IEI causing copy number variants. **a** homozygous deletions of *ICOS* as observed from WES data. **b** heterozygous deletion of *TNFAIP3* as observed from WES and SNP array. **c** heterozygous deletions of 22q11.2 as observed from WES and SNP array

Small deletions with breakpoints located within IEI-associated genes were identified in six samples. Homozygous intragenic deletions affecting exons 2 and 3 of *ICOS* with a size of around 1.2 kb were observed in two samples (Fig. 1a; patient-005 and patient-060). Please note that patient-005 has been previously reported in 2003 and served as a blinded control in this study to verify reliable detection by NGS of this founder variant causing ICOS deficiency [23]. Patient-063 presented a heterozygous 2.9 kb deletion affecting exons 2–6 of *UBE2T* (out of 7 exons; NM_014176.4), including loss of the transcription start codon located within exon 2. Patient-047 presented a heterozygous 17.6 kb deletion affecting the last exon of *CORO1A* (exon 12 out of 12 exons; NM_001193333.3) causing a loss of amino acids 428–461 including the C-terminal coiled-coil leucine zipper domain. This deletion included further genes expanding to *SULT1A3*. In patient-141, a heterozygous 6.1 kb deletion of *FANCA* exons 6–8 (out of 43 exons; NM_000135.4) was observed, indicating an in-frame loss of amino acids 175–264. A heterozygous 6.2 kb deletion of *TYK2* exons 4–9 (out of 25 exons; NM_003331.5), causing a potential out-of-frame deletion of amino acids 65–456, was observed in patient-008.

Larger deletions affecting complete genes associated with IEI were observed in five samples. A monoallelic loss of *TNFAIP3* was caused by a heterozygous 59.9 kb deletion in patient-191 (Fig. 1b). Two samples showed heterozygous deletions affecting chromosome 22q11.2, which is an autosomal dominantly inherited gene defect commonly observed in PID (Fig. 1c). Patient-017 presented a 2.6 Mb deletion of this locus including the gene *TBX1*, whereas patient-180 carried a smaller deletion of 740.0 kb excluding *TBX1*. The largest CNV identified in this cohort was a monoallelic 8.7 Mb deletion covering more than 60 genes, including *ATM* (patient-058). In patient-122, *CD19* and *LAT* were part of a large heterozygous 167.8 kb deletion affecting a total of nine protein-coding genes.

### Genomic Structure Copy Number Gains

Copy number gains affecting a gene from the IEI classification list were observed in nine samples (Table 1). Optical genome mapping was carried out to determine if duplications were in tandem or translocated to a distant region. Four samples showed TDs larger than 100 kb (Fig. S2; patient-081, patient-083, patient-074, patient-090). The IEI-associated genes *TRAC*, *SLC7A7*, and *CEBPE* were part of a 591 kb TD observed for patient-083. In synopsis of exome, array, and mapping data, breakpoints can be identified to be directly upstream of *TRAC* and within *SLC7A8*. Patient-090 and patient-074 displayed duplications containing entire IEI genes as well. For patient-090 *MEFV* was duplicated in tandem with breakpoints located 70 kb upstream and 80 kb downstream of the gene. Patient-074 showed a 739 kb TD including *CORO1A*. In patient-081, a 397 kb TD affected exons 1–35 of *LRBA* as well as exons 10–21 of the neighboring gene *SH3D19* (NM_001009555.4).

Patient-171 showed a 9.3 kb duplication of *TRIM22* exons 1 to 4 (out of 8 exons). OGM indicates that the duplicated material has been inserted in tandem affecting the region harboring the potential promoter of *TRIM22* and the 5′ region of the neighboring gene *TRIM5*. There is no information on the orientation of the inserted duplication, as there were no fluorescent markers present in the duplicated material. Patient-160 showed an 11 kb duplication in array analysis, including the IEI gene *BRIP1* exons 1–3 (out of 20 exons) and part of the 3′ region of the neighboring gene *INTS2*. OGM detected a 14 kb insertion into the same region and interpretation as a TD, with a breakpoint in *INTS2*, was supported by presence of three fluorescent markers. For patient-149, a duplication observed in SNP array affecting the terminal exons 4–10 of *IKBKG* was represented as a 12 kb insertion located to the same position.

No material for OGM was available for two samples. Patient-094 exhibited an 18 kb duplication affecting *PSTPIP1* exons 2–12 (out of 15 exons), and patient-114 displayed a large 743 kb duplication containing the IEI gene *CYBA*.

### Sequence Variants in IEI-Associated Genes

Patients carrying a CNV were analyzed for sequence variants. We identified SNVs likely to be involved in the expression of the phenotypes observed in patient-063, patient-094, and patient-074 (Table 1). Patient-063 carried two heterozygous SNVs affecting *DOCK2*, a pathogenic frameshift variant and a likely pathogenic missense variant; however, samples of the parents were unavailable to confirm compound heterozygosity. Patient-094 carried a heterozygous hypermorphic and pathogenic SNV affecting the donor splice site of *PIK3R1* intron 11, previously reported in the literature (MIM 171,833#0008). A heterozygous pathogenic missense SNV affecting *NFkB1* was observed in patient-074. Additional variants with an unclear clinical relevance were identified in patient-171 (missense variant in *NFkB2*), and patient-083 (nonsense variant in *NLRC4*). All sequence variants were validated using Sanger sequencing.

## Discussion

The contribution of rare CNVs to genetic driven diseases has been well recognized in recent years and identification of causative CNVs is being promoted by technical and bioinformatic advances as well as the availability of large population based CNV data sets [24]. CNV analysis promises to improve the yield of molecular diagnoses as well as to explain phenotypic heterogeneity potentially caused by different types of aberrations targeting the same gene (e.g. SNV versus CNV) [7]. Accordingly, a spectrum of different CNVs affecting different genes has also been reported in IEI. In this study, we aimed to identify further relevant CNVs by evaluating WES data of a large cohort of patients with a suspected IEI using the ClinCNV algorithm.

Our analysis identified 20 CNVs in 20 different patients. Within these 20 patients, sequence analysis of IEI associated genes identified six possibly relevant SNVs in five samples. The clinical phenotypes of eight patients can be plausibly explained by these CNVs or SNVs, which are summarized in the upper part of Table 1 “samples with molecular diagnosis” and will be discussed in the following. The remaining twelve patients presented CNVs and SNVs either with a less distinct correlation to the observed phenotypes or presented a heterozygous event in a gene with a so far known autosomal recessive inheritance. These patients are summarized in the lower part of Table 1 “samples with uncertain molecular diagnosis” and are discussed in the online supplement.

Conclusive molecular diagnoses could be made by our analysis for patient-005 and patient-060. Both patients carried a homozygous pathogenic *ICOS* deletion, representing a known founder variant [23]. The diagnosis of ICOS deficiency is consistent with symptoms observed for both patients indicating perturbed T- and B-cell development, as well as signs of recurrent infections [25]. Likewise, heterozygous deletion of *TNFAIP3* in patient-191 is another conclusive molecular diagnosis. This gene is described with an autosomal dominant inheritance in IEI, and ACMG guidelines allow classification of this CNV as pathogenic. Clinical manifestations observed in the carrier include rosacea, lymphadenopathy, splenomegaly, pulmonary emphysema, and chronic sinusitis. The protein A20, encoded by *TNFAIP3*, is a negative regulator of the TNF-α/NF-κB signaling pathway, and haploinsufficiency of A20 has been described to cause autoinflammatory disease in a number of cases [26–28]. Deletions of chromosomal region 22q11.2, as seen in two cases (patient-017 and patient-180) of our study, have been described before and are amongst the most common gene defects in IEI [5]. A cluster of low-copy repeats (LCR22-A to LCR22-H) makes this region prone to deletions and duplications [29]. Dependent on the LCRs involved, CNVs show recurrent sizes. Patient-017 showed a deletion of almost 2.6 Mb with breakpoints indicated to involve LCR22-A and LCR22-D (dosage ID: ISCA-37446). This deletion has been described to cause DiGeorge syndrome, and clinical manifestations observed in our patient including recurrent infections of the upper respiratory tract, recurrent pneumonia, and seizures seem to be accountable to this deletion. Our second patient (patient-180) carried a smaller deletion of 704 kb at chromosome 22q11.2 with breakpoints potentially involving LCR22-B and LCR22-D. However, atypical breakpoints outside of LCRs are possible and cannot be ruled out [29]. This deletion does not affect the DGS critical genes *HIRA* and *TBX1*; however, immune deficiency and recurrent infections have been described for this region. Interestingly, T cell count was better in patient-180 with the non-*TBX1* inclusive deletion compared to patient-017 with *TBX1* deletion, which is in line with other observations in the literature [30].

Patient-063, patient-094, and patient-074 showed CNVs targeting functionally relevant genes with an established role in immune regulation. However, sequence analysis for these samples identified additional SNVs, which can reasonably explain the clinical phenotypes. Patient-063 carried two heterozygous and likely relevant SNVs affecting *DOCK2*. DOCK2 deficiency is a rare combined immunodeficiency characterized by early-onset of recurrent, invasive viral and bacterial infections associated with T and B cell lymphopenia. The disease has a poor prognosis without bone marrow transplantation. In agreement, patient-063 presented with early onset and severe EBV infection reduced level of naïve CD4 T cells and death at age 17. Additionally, the patient carried an intragenic *UBE2T* deletion. UBE2T is a ubiquitin ligase and a component of the Fanconi anemia pathway (FANCT, MIM 616,435) [31]. The heterozygous deletion identified in our patient affects the majority of the coding exons including the translation start site. For the reason that Fanconi anemia is an autosomal recessive disease, the contribution of this deletion remains speculative, yet occurrence of lymphoma in the patient might fall in the spectrum of Fanconi anemia [32]. Patient-094 presented an intragenic duplication of *PSTPIP1* and notably this patient carried a pathogenic gain-of-function variant in *PIK3R1* (MIM 616,005#0008), which is highly likely to contribute to the clinical features observed in this patient, including splenomegaly, severe EBV infection, and expansion of transitional B cells. Pathogenic variants in *PSTPIP1* (proline-serine-threonine phosphatase interacting protein 1) cause PAPA syndrome an autosomal dominant autoinflammatory disorder characterized by pyogenic arthritis, pyoderma gangrenosum, and severe cystic acne (MIM 604,416) [33]. However, phenotype data available for this patient does not fit well to PAPA syndrome. Assuming an integration in tandem, as indicated by OGM, this internal duplication would introduce a frame-shift and most likely a loss of function. The discrepancy in phenotypes might be then explained by the different functional consequences of a large duplication compared to previously reported missense variants in PAPA syndrome [34]. Sequence analysis of patient-074 identified a pathogenic missense SNV in *NFKB1*, which has recently been shown to be a loss of function variant and thus explains the clinical phenotype [35]. The spectrum of clinical features observed in our patient was extensive and severe including but not limited to impaired immune cell function, severe recurrent viral infections from early childhood to adulthood, and manifestation of long-term conditions, amongst them hepatomegaly, lymphadenopathy, splenomegaly, granulomatosis, interstitial lung disease, enteropathy, arthritis, and alopecia. Additionally, this patient carried a pathogenic TD of 576 kb compatible with the 16p11.2 duplication syndrome (dosage ID: ISCA-37400). No immunological defects have been reported for this aberration in the MIM database so far (MIM 614,671), and the patient did not present previously described features of speech/language delay or cognitive impairment. The duplicated region contained *CORO1A*, and at this point, it is unclear if and how function of this gene associated with IEI might be impaired by this duplication.

This study carries limitations that have to be addressed. Exome sequencing data revealed a tendency for false-positive CNV calls, which made an independent validation by SNP array necessary. Interestingly, characteristics of negatively validated CNVs (e.g., deletion-duplication ratio, copy number state, or size) did not show obvious differences from positively validated CNVs, thus preventing prediction of false positive CNVs. As a general remark, special attention should be paid to dense probe coverage of IEI associated genes when establishing NGS- or array-based CNV analysis in routine diagnostics. Another limitation is that whole blood and PBMCs are common source materials in many studies, including ours, as they are easily accessible and ensure a sufficient quantity of isolated DNA. However, this bulk analysis of DNA from different cell types can impede to detect somatic mosaicism, which can be a relevant cause of immunodeficiency [36]. Additionally, bulk analysis can cause misinterpretation of zygosity; e.g., heterozygous deletions might be in fact homozygous deletions present in a small fraction of cells only. Furthermore, analysis of the parents would have been helpful to evaluate if aberrations occurred de novo or were inherited. This inheritance information would have been of special value for the twelve samples carrying CNVs or SNVs of uncertain significance (Table 1 and online supplementary discussion). One highly relevant limitation is that a deeper follow-up on the functional consequences was beyond the scope of this study and effects of most CNVs and SNVs could only be discussed theoretically. Particularly predicting the functional consequences of duplications is challenging and strongly dependent on breakpoint positions within the gene locus, triplosensitivity of genes (which is often unknown), and on positional effects. However, we believe reporting these results is valuable to shed further light on how phenotypic heterogeneity in IEI might be shaped by genetic aberrations other than well-explored sequence variants.

In conclusion, our copy number analysis from WES data identified clinically meaningful CNVs in 2.6% of patients, including deletions affecting *ICOS*, *TNFAIP3*, and 22q11.2. Another 7.9% of patients carried potentially relevant CNVs with a less distinct correlation to the observed phenotypes and for which a follow-up by functional analyses is indicated. This study points out that careful evaluation of CNVs from exome data, often generated solely for SNV analysis in routine diagnostic, has potential to increase the diagnostic yield substantially. In the end, refining clinical diagnoses to molecular diagnoses will impact the treatment of the patients and will allow a more comprehensive genetic counseling of patients and their relatives.

## Supplementary Information

Below is the link to the electronic supplementary material.

Supplementary file1 (DOCX 593 kb)

## Data Availability

The datasets generated during and/or analyzed during the current study are available from the corresponding author on reasonable request.

## Abbreviations

ACMG: American College of Medical Genetics
ClinGen: Clinical Genome Resource at https://www.clinicalgenome.org
CNV: Copy number variant
CVID: Common variable immunodeficiency
DGV: Database of Genomic Variants
gnomAD: Genome aggregation database
IEI: Inborn errors of immunity
IGV: Integrative Genomics Viewer
IUIS: International Union of Immunological Societies
LCR: Low-copy repeat
MAF: Minor allele frequency
megSAP: Medical genetics Sequence Analysis Pipeline
NGS: Next-generation sequencing
OGM: Optical genome mapping
PID: Primary immunodeficiency
SNV: Single nucleotide variant
TD: Tandem duplication
WES: Whole exome sequencing
