# Supplementary material for: Copy Number Analysis in a Large Cohort Suggestive of Inborn Errors of Immunity Indicates a Wide Spectrum of Relevant Chromosomal Losses and Gains
Source: J Clin Immunol. 2022 Apr 29;42(5):1083–92. doi: 10.1007/s10875-022-01276-8 (PMC9402522; doi:10.1007/s10875-022-01276-8)
Supplement: Supplementary file 1 — Supplementary file1 (DOCX 593 kb) [file 10875_2022_1276_MOESM1_ESM.docx]

**Copy number analysis in a large cohort suggestive of inborn errors of immunity indicates a wide spectrum of relevant chromosomal losses and gains**

Rensheng Wan^1,*^, Maximilian Schieck^1,2*^, Andrés Caballero-Oteyza^3^, Winfried Hofmann^1^, Alexis Virgil Cochino^4^, Anna Shcherbina^5^, Roya Sherkat^6^, Clarisse Wache-Mainier^7^, Anita Fernandez^7^, Marc Sultan^7^, Thomas Illig^1,2,8^, Bodo Grimbacher^2,3,9,10,11#^, Michele Proietti^2,3,12#^, Doris Steinemann^1,2#^

^1^ Department of Human Genetics, Hannover Medical School, Hannover, Germany

^2^ RESIST-Cluster of Excellence 2155, Hannover Medical School, Hannover, Germany

^3^ Institute for Immunodeficiency, Center for Chronic Immunodeficiency, Medical Center, Faculty of Medicine, Albert-Ludwigs-University, Freiburg, Germany

^4^ Institute for Mother and Child Health, Bucharest, Romania

^5^ Dmitry Rogachev National Medical and Research Center for Pediatric Hematology, Oncology, Immunology, Moscow, Russia

^6^ Acquired Immunodeficiency Research Center, Isfahan University of Medical Sciences, Isfahan, Iran

^7^ Novartis Institutes for BioMedical Research, Novartis Pharma AG, Basel, Switzerland

^8^ Hannover Unified Biobank, Hannover Medical School, Hannover, Germany

^9^ Clinic for Rheumatology and Clinical Immunology, Center for Chronic Immunodeficiency (CCI), Medical Center, Faculty of Medicine, Albert-Ludwigs-University of Freiburg, Germany

^10^ DZIF – German Center for Infection Research, Satellite Center Freiburg, Germany

^11^ CIBSS – Centre for Integrative Biological Signalling Studies, Albert-Ludwigs University, Freiburg, Germany

^12^ Department of Rheumatology and Clinical Immunology, Hannover Medical School, Hannover, Germany

^*^ and ^#^ equal contribution

**ONLINE SUPPLEMENT**

In the supplementary discussion we report and discuss CNVs and SNVs of uncertain significance (summarized in the lower part of Tab.1 “samples with uncertain molecular diagnosis”). These variants either showed less correlation to the clinical phenotype or presented a monoallelic event in a gene with a so far known autosomal recessive inheritance.

**Deletions**

Patient-058

The largest CNV, a heterozygous deletion of almost 9 Mb at 11q22.1q22.3, was observed in patient-058. ACMG guidelines allow classification of this CNV as pathogenic. According to the ClinGen database, this region harbors 48 protein-coding genes including *ATM,* previously associated with IEI. More than half of the patients with ataxia telangiectasia carrying biallelic *ATM* variants show a clinical history of susceptibility to infections [1]. In line with previous reports, clinical features of this patient include recurrent lower respiratory tract infections, and decreased IgA and IgG levels [2]. However, we did not detect a second variant (SNV nor CNV) on the other *ATM* allele. At this point it remains unclear if relevant variants, e.g. in non-coding *ATM* regions, are missed by our exome-based approach or if the large deletion itself has a functional effect causing the clinical presentation, including disturbed generation of adaptive immunological diversity [3].

Patient-122

Patient-122 presented a deletion of 168 kb at 16p11.2 including *CD19* and *LAT*. For both genes an autosomal recessive inheritance in IEI has been described. Again, our analysis indicated no aberration on the second allele. The deleted region corresponds to the known chromosome 16p11.2 deletion syndrome. ClinGen reports this region as susceptible to haploinsufficiency and associated key clinical features are developmental delay and early onset obesity (dosage ID: ISCA-37486; ACMG classification “pathogenic”), which were not observed for patient-122. To what extent a possible haploinsufficiency of *CD19* and *LAT* could explain the clinical symptoms of the patient (e.g. hypogammaglobulinemia) remains open.

Besides large deletions affecting whole genes, we identified a number of microdeletions smaller than 20 kb affecting only parts of IEI-associated genes.

Patient-047

Patient-047 carried a small 17.6 kb deletion affecting the last exon of *CORO1A.* This should lead to a functional inactivation due to loss of the C-terminal coiled-coil leucine zipper oligomerization domain of CORO1A (deletion of aa 428-461; NP_001180262.1). The C-terminal CORO1A domains are relevant to T-cell survival and function and some of the patient’s clinical features are in line with previously reported cases [4], including abnormal lymphocyte proliferation, increased proportion of effector memory CD8^+^ T-cells, and reduced proportion of naive CD4^+^ T-cells.

Patient-008

An intragenic deletion of *TYK2* causing a potential frame-shift, is compatible with clinical symptoms observed for patient-008, including onset of symptoms in childhood, recurrent respiratory infections, and increased susceptibility to virus (MIM 611521). Additionally, this patient has evidence of autoimmune disease (e.g. positive antinuclear antibody (ANA) test), and a strong link between *TYK2* variants and autoimmune diseases has been reviewed recently [5].

Patient-141

Small deletions of *FANCA* have been frequently reported in PID and patient-141 presented an intragenic *FANCA* deletion leading to a potential in-frame loss of aa 175-265 [6, 7]. This substantial loss of coding sequence is likely to impair protein function. FANCA has been suggested to play a role in primary diversification of immunoglobulins. It is therefore very well possible that the deletion of *FANCA* is causative for at least some of the observed clinical features in patient-141, including decreased Ig levels (IgA, IgM, IgG1, IgG4) and decreased proportions of plasmablasts and switched memory B cells [8].

**Duplications**

We identified a number of duplications affecting IEI-associated genes (Fig.S2). Three patients presented gains of chromosomal material affecting the entire coding region of IEI-associated genes.

Patient-083

Patient-083, with an early onset of CID at age 2.2 years, carried a large 600 kb duplication at 14q11.2 affecting more than 20 genes including *TRAC*, *SLC7A7* and *CEBPE*. Dosage effects of duplicated genes might be responsible for the clinical presentation of recurrent and severe virus infections in this patient. Besides, this TD brings one copy of *CEBPE* in close vicinity of approx. less than 30 kb to *TRAC* (T cell receptor alpha chain constant) (Fig.S2). A broad influence on the inflammasome and interferome is known for the transcription factor C/EBPε encoded by *CEBPE* [9]. If this rearrangement leads to an altered *CEBPE* expression is unclear. Unfortunately, no suitable material was available to test for aberrant C/EBPε levels specifically in T cells, which might be responsible for the low T cell count observed in this patient due to pro-apoptotic features of C/EBPε [10]. A heterozygous pathogenic nonsense variant in *NLRC4* adds more complexity to this case. However, the patient shows no phenotype suspective of NLRC4 inflammasomopathy, usually caused by missense variants of *NLRC4*, and the nonsense variant observed in our patient might lead to different phenotypic consequences [11].

Patient-090

Patient-090 carried a 103 kb TD encompassing the *MEFV* gene. The patient did not present typical symptoms of familial Mediterranean fever caused by defects of *MEFV* (MIM 608107), but variability of symptoms as well as subclinical phenotypes have been suggested [12]. The patient carried no other relevant SNVs or CNVs and further analysis on *MEFV* duplications in IEI could be considered. However, the ClinGen consortium reports no evidence for triplosensitivity of *MEFV* (dosage ID: ISCA-24503) and a genome-wide analysis beyond IEI-associated genes is considered for this specific case.

Patient-114

A 743 kb duplication of chromosome 16q24.2 in patient-114 was the largest chromosomal gain identified in this study. *CYBA* was part of the duplicated region, however at this point it is not clear how *CYBA* function might be affected by the duplication. Yet, defects of *CYBA* are known to cause chronic granulomatous disease 4 (MIM 608508) and in line with this the patient presented pulmonary granulomatosis at age 23 years [13].

Four patients carried duplications with breakpoints potentially located within IEI-associated genes. Duplications either affected 5’ gene regions (patient-081, patient-171, and patient-160) or the 3’ gene region (patient-149).

Patient-081

Analysis of patient-081 with OGM showed a TD, meaning that one *LRBA* copy is putatively left unaffected and a putative fusion of *LRBA* to *SH3D19*, consisting of the *LRBA* promoter region, *LRBA* exons 1-35 and *SH3D19* exons 10-21. The putative fusion protein product would consist of the first 1882 out of 2863 LRBA amino acids (approx. 66% of the LRBA coding sequence*)* with a putative premature stop codon at position 1905. This would likely cause nonsense-mediated mRNA decay (NMD). However, breakpoints are not resolved at sequence-level in our study and NMD does not necessarily need to be activated.

Patient-171

In patient-171 a 9.3 kb duplication of *TRIM22* exons 1 to 4 was indicated to be inserted in tandem. This TD potentially affects the region harboring the potential promoter of *TRIM22* and the 5’ region of the neighboring gene *TRIM5*. TRIM22 has an antiviral function and is involved in activation of interferon-beta signaling and nuclear factor-κB [14, 15]. Additionally, this patient carries a rare missense VUS in *NFKB2* (p.Arg261Trp). Based on this data, one can only speculate which of the two aberrations are causing the clinical phenotype (Tab.S2) or if additive effects of the *TRIM22* TD and the *NFKB2* VUS persist in patient-171.

Patient-160

The third 5’ gene region duplication identified in this study was a TD of *BRIP1* exons 1-3. This gene is associated with Fanconi anemia (MIM 609054) and perturbed humoral immunity observed in patient-160, including hypogammaglobulinemia and decreased proportion of plasmablasts, might be explained by a defect in the Fanconi anemia pathway.

Patient-149

A TD of *IKBKG* exons 4-10 in patient-149 was the only duplication of a 3’ gene region of an IEI-associated gene in this study. No other variants were observed for this patient, making *IKBKG* a promising explanation for hypogammaglobulinemia alongside with decreased proportion of plasmablasts observed in this patient (MIM 300636).

**Fig.S1: Analysis strategy for the detection and validation of CNVs and SNVs in patients with an IEI**

**
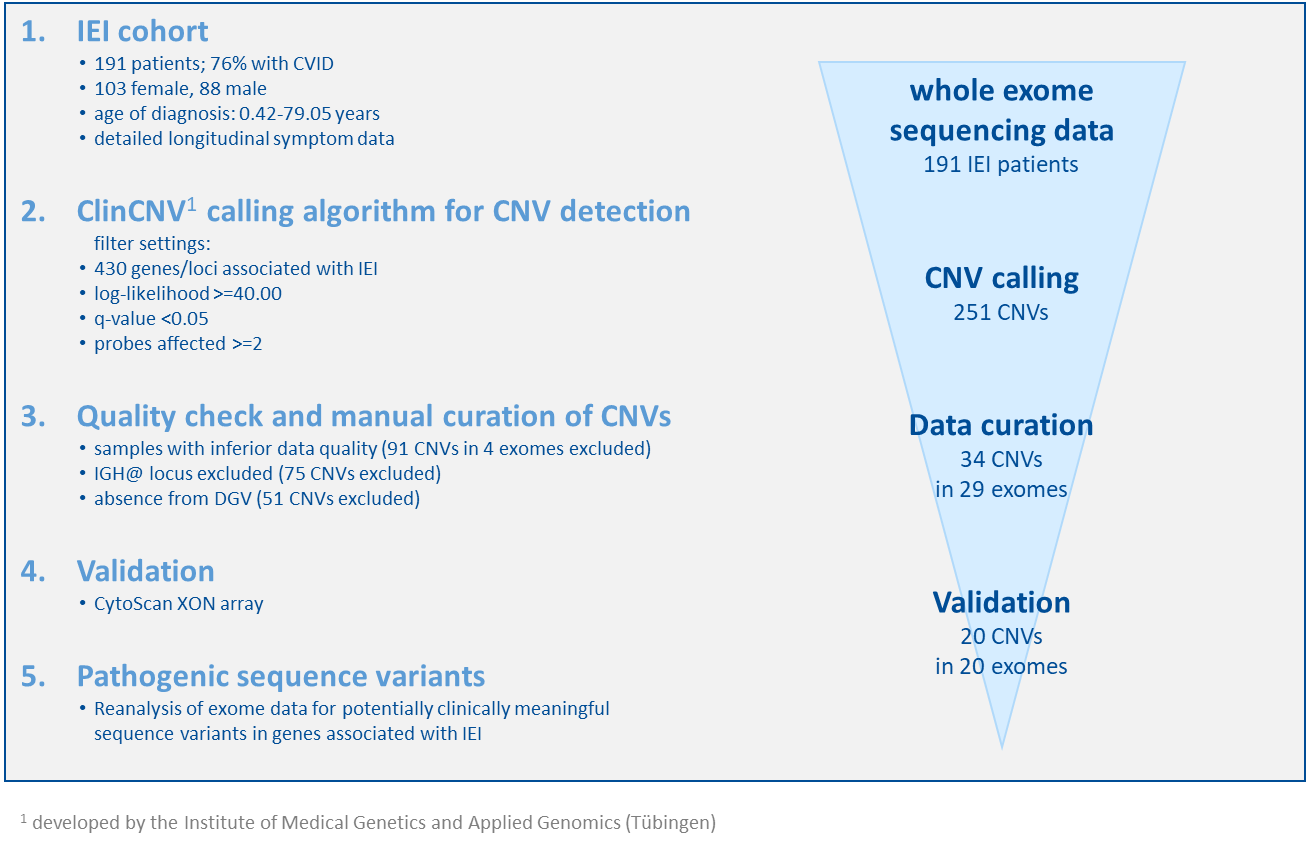
**

**Fig.S2: Confirmation of duplications using optical genome mapping.** OGM analysis showed that duplications identified from WES data were in tandem (patient-074, patient-081, patient-083, patient-090, and patient-160) or to be likely in tandem (patient-149, and patient-171).

**
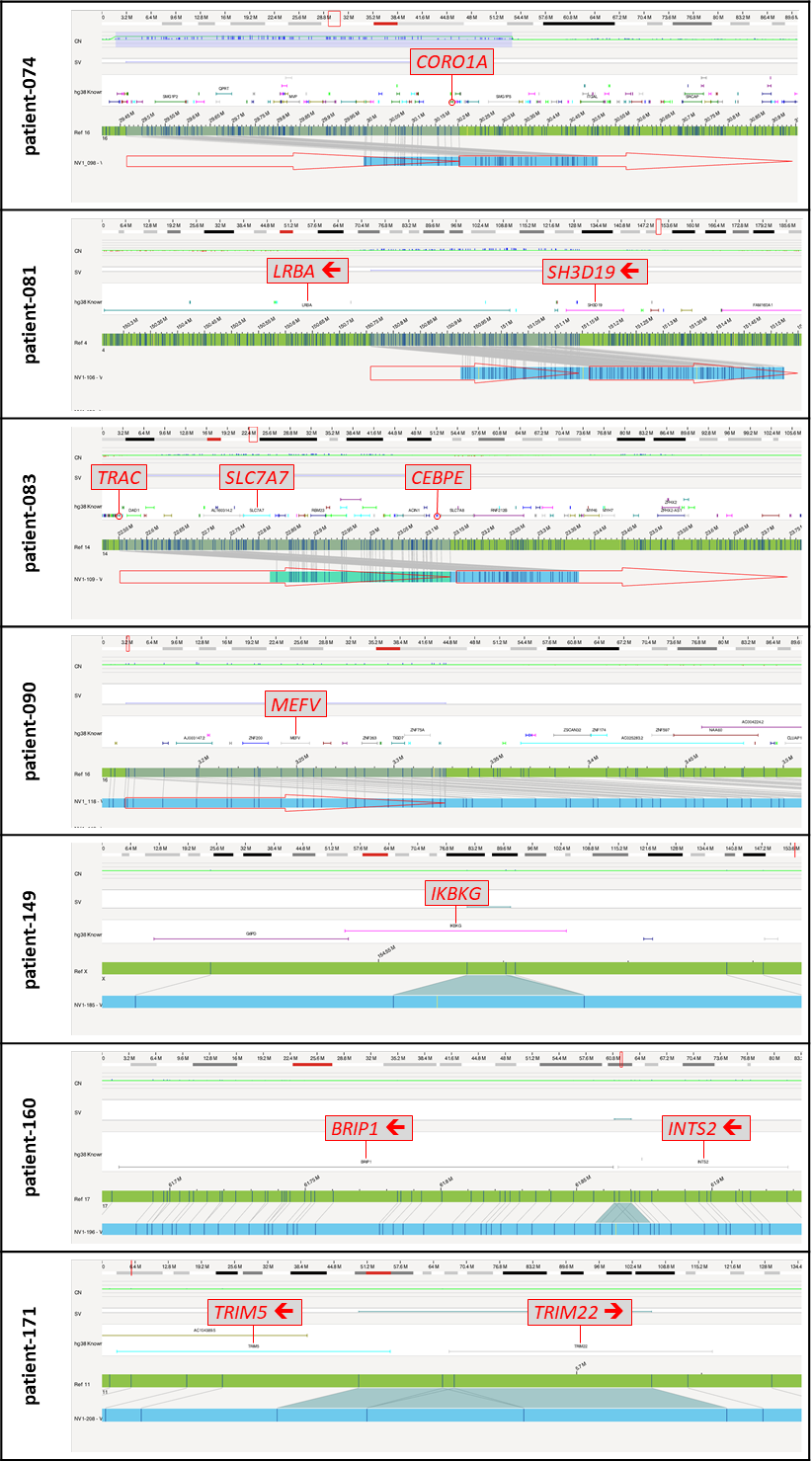
**

**Tab.S1: Cohort of patients with a suspected IEI**

**Tab.S2: Clinical phenotypes of patients carrying a validated CNV affecting an IEI-associated gene.**

**REFERENCES**

1. Staples ER, McDermott EM, Reiman A, Byrd PJ, Ritchie S, Taylor AM, et al. Immunodeficiency in ataxia telangiectasia is correlated strongly with the presence of two null mutations in the ataxia telangiectasia mutated gene. Clin Exp Immunol. 2008;153(2):214-20.

2. Bousfiha A, Jeddane L, Picard C, Al-Herz W, Ailal F, Chatila T, et al. Human Inborn Errors of Immunity: 2019 Update of the IUIS Phenotypical Classification. J Clin Immunol. 2020;40(1):66-81.

3. Weitering TJ, Takada S, Weemaes CMR, van Schouwenburg PA, van der Burg M. ATM: Translating the DNA Damage Response to Adaptive Immunity. Trends Immunol. 2021;42(4):350-65.

4. Yee CS, Massaad MJ, Bainter W, Ohsumi TK, Foger N, Chan AC, et al. Recurrent viral infections associated with a homozygous CORO1A mutation that disrupts oligomerization and cytoskeletal association. J Allergy Clin Immunol. 2016;137(3):879-88 e2.

5. Pellenz FM, Dieter C, Lemos NE, Bauer AC, Souza BM, Crispim D. Association of TYK2 polymorphisms with autoimmune diseases: A comprehensive and updated systematic review with meta-analysis. Genet Mol Biol. 2021;44(2):e20200425.

6. Stray-Pedersen A, Sorte HS, Samarakoon P, Gambin T, Chinn IK, Coban Akdemir ZH, et al. Primary immunodeficiency diseases: Genomic approaches delineate heterogeneous Mendelian disorders. J Allergy Clin Immunol. 2017;139(1):232-45.

7. Dyer L, Li X, Denton J, Jones B, Liston E, Hilton D, et al. Gene dosage defects in primary immunodeficiencies and related disorders: a pilot study. Journal of Translational Genetics and Genomics. 2017;1:23-7.

8. Nguyen TV, Pawlikowska P, Firlej V, Rosselli F, Aoufouchi S. V(D)J recombination process and the Pre-B to immature B-cells transition are altered in Fanca(-/-) mice. Sci Rep. 2016;6:36906.

9. Goos H, Fogarty CL, Sahu B, Plagnol V, Rajamaki K, Nurmi K, et al. Gain-of-function CEBPE mutation causes noncanonical autoinflammatory inflammasomopathy. J Allergy Clin Immunol. 2019;144(5):1364-76.

10. Nakajima H, Watanabe N, Shibata F, Kitamura T, Ikeda Y, Handa M. N-terminal region of CCAAT/enhancer-binding protein epsilon is critical for cell cycle arrest, apoptosis, and functional maturation during myeloid differentiation. J Biol Chem. 2006;281(20):14494-502.

11. Romberg N, Vogel TP, Canna SW. NLRC4 inflammasomopathies. Curr Opin Allergy Clin Immunol. 2017;17(6):398-404.

12. Kallinich T, Orak B, Wittkowski H. [Role of genetics in familial Mediterranean fever]. Z Rheumatol. 2017;76(4):303-12.

13. Salvator H, Mahlaoui N, Catherinot E, Rivaud E, Pilmis B, Borie R, et al. Pulmonary manifestations in adult patients with chronic granulomatous disease. Eur Respir J. 2015;45(6):1613-23.

14. Reddi TS, Merkl PE, Lim SY, Letvin NL, Knipe DM. Tripartite Motif 22 (TRIM22) protein restricts herpes simplex virus 1 by epigenetic silencing of viral immediate-early genes. PLoS Pathog. 2021;17(2):e1009281.

15. Li Q, Lee CH, Peters LA, Mastropaolo LA, Thoeni C, Elkadri A, et al. Variants in TRIM22 That Affect NOD2 Signaling Are Associated With Very-Early-Onset Inflammatory Bowel Disease. Gastroenterology. 2016;150(5):1196-207.
